# *Mentha arvensis* Essential Oil Exerts Anti-Inflammatory in LPS-Stimulated Inflammatory Responses via Inhibition of ERK/NF-κB Signaling Pathway and Anti-Atopic Dermatitis-like Effects in 2,4-Dinitrochlorobezene-Induced BALB/c Mice

**DOI:** 10.3390/antiox10121941

**Published:** 2021-12-03

**Authors:** So-Yeon Kim, Sang-Deok Han, Minju Kim, Tamanna Jahan Mony, Eun-Seok Lee, Kyeong-Min Kim, Seung-Hyuk Choi, Sun Hee Hong, Ji Woong Choi, Se Jin Park

**Affiliations:** 1 Department of Food Biotechnology and Environmental Science, School of Natural Resources and Environmental Sciences, Kangwon National University, Chuncheon 24341, Korea; ykims95@kangwon.ac.kr (S.-Y.K.); 202016097@kangwon.ac.kr (S.-D.H.); scent@kangwon.ac.kr (M.K.); dmstjr0806@kangwon.ac.kr (E.-S.L.); kasbai@kangwon.ac.kr (K.-M.K.); chltmdgur96@kangwon.ac.kr (S.-H.C.); 2 Agriculture and Life Science Research Institute, Kangwon National University, Chuncheon 24341, Korea; tjmonycvasu@gmail.com; 3 School of Applied Science in Natural Resources & Environment, Hankyong National University, Anseong 17579, Korea; shhong@hknu.ac.kr; 4 College of Pharmacy, Gachon University, Incheon 21936, Korea

**Keywords:** *Mentha arvensis*, atopic dermatitis, inflammation, inflammatory cytokine, nuclear factor-kappa B

## Abstract

The mechanism of atopic dermatitis (AD) is modulated by the release of cytokines and chemokines through the mitogen-activated protein kinase (MAPK)/nuclear factor-kappa B (NF-κB) signaling pathway. Topical steroids are used to treat AD, but some people need safer anti-inflammatory drugs to avoid side effects. *Mentha arvensis* has been used as a herbal plant with medicinal properties, but its anti-inflammatory effects have not been elucidated in an AD model. In this study, we investigated the anti-inflammatory effects of *M. arvensis* essential oil (MAEO) and its underlying molecular mechanism in lipopolysaccharide (LPS)-stimulated RAW 264.7 macrophages and HaCaT cells (human epidermal keratinocyte). Additionally, we examined the ameliorating effects of the MAEO in a dinitrochlorobenzene (DNCB)-induced murine model of AD. We found, in both RAW 264.7 cells and HaCaT cells, MAEO inhibited LPS-stimulated inflammatory mediators such as nitric oxide (NO) and prostaglandin E_2_ and proinflammatory cytokines, including IL-1β and IL-6, due to the suppression of COX-2 and iNOS expression. In LPS-stimulated macrophages, we also observed that MAEO inhibited the phosphorylation of ERK and P65. Furthermore, MAEO treatment attenuated AD symptoms, including the dermatitis score, ear thickness, epidermal thickness and infiltration of mast cells, in a DNCB-induced animal model of AD. Overall, our findings suggest that MAEO exerts anti-inflammatory and anti-atopic dermatitis effects via inhibition of the ERK/NF-κB signaling pathway.

## 1. Introduction

Atopic dermatitis (AD) is a chronic inflammatory skin disease with complex interactions between the environment and the immune system via the epidermal barrier [1]. Several recent studies have shown that an increasing number of patients are suffering from AD due to urbanization and industrialization, and the prevalence of children with AD reached 25.9% in 2017 [2,3,4]. AD is caused by an abnormal immune response of activated immune cells and skin cells characterized by itching, dryness and skin dysfunction [5]. In general, mast cells that produce various cytokines, including IL-4 and IL-13, in response to various stimuli are found in AD skin lesions [6]. Therefore, regulating cytokine production in immune cells and skin cells might be an effective therapeutic strategy for AD. Relief of itching has been considered as a treatment of AD. However, a histamine receptor antagonist (antihistamine) was not sufficient to suppress itching in AD patients [7]. Another alternative, steroids, cannot be used in long-term treatment due to side effects such as the risk of thinning and weakening the skin barrier [8]. Therefore, additional research is necessary to develop safer treatments.

Nuclear factor-kappa B (NF-κB) is an important mediator of inflammatory and immune responses in AD. Since NF-κB is regulated by mitogen-activated protein kinases (MAPKs) and it activates gene transcription in the nucleus, it plays a pivotal role in the regulation of the inflammatory response [9]. NF-κB enhances the secretion of proinflammatory cytokines including, interleukin (IL)-6, IL-1β and tumor necrosis factor-α (TNF-α). Cytokines and chemokines released by the activation of the MAPK/NF-κB signaling pathway are important for immune cell inflammation and skin barrier function [10]. Nitric oxide (NO) and prostaglandin E_2_ (PGE_2_), which are synthesized by inducible nitric oxide synthase (iNOS) and cyclooxygenase-2 (COX-2), respectively, are major mediators of the inflammatory response such as pain, edema, dysfunction, fever and the movement of immune cells [11,12,13]. Thus, cytokines, such as TNF-α and interleukins (ILs), are induced by the overproduction of NO and promote the inflammatory response and tissue damage.

*Mentha arvensis*, known as mint, is a species of flowering plant in the mint family Lamiaceae. Mentha species are generally grown in a circumboreal distribution and are native to Western and Central Asia, Europe and North America [14]. Young stems are used in food due to the fragrance of their flavoring agents in chewing gum, candies, drinks, cosmetics and tobacco [15]. Whole plants have been commonly used as Chinese medicines with many medicinal properties, such as anti-inflammatory and antioxidative activities [16]. Cultivation of aromatic plants to obtain essential oils has become increasingly popular [17]. Among the many aromatic species, *M. arvensis* has great economic importance because of its large number of applications in different sectors of the pharmaceutical industry [18]. However, the anti-inflammatory and anti-atopic effects of MAEO and its underlying molecular mechanisms have not been fully elucidated until now. Thus, the objective of this study was to evaluate the anti-inflammatory properties of MAEO and its molecular mechanism of action in LPS-stimulated RAW 264.7 murine macrophages and HaCaT human keratinocytes. In addition, the anti-atopic dermatitis effect of MAEO was investigated using the 2,4-dinitrochloro benzene (DNCB)-induced murine model of AD.

## 2. Materials and Methods

### 2.1. Animals

The animal experiments were performed according to the guidelines for animal experiments of the Institutional Animal Care and Use Committee (IACUC) of the Laboratory Animal Research Center at Kangwon National University, Korea (KW-200122-2). BALB/c mice (6-weeks-old) were purchased from Orient Bio (Seongnam, Korea). Each cage housed five mice at a controlled (21–25 °C), 12 h light–dark cycle, and relative humidity (45–65%). Food and water were available *ad libitum*.

### 2.2. Materials

*Mentha arvensis* (*M. arvensis*) (DJU20171135) was purchased from Jecheon Herb, Chungbuk Herbal Medicine Farming Association (Jecheon, Korea). Dulbecco’s modified Eagle’s medium (DMEM), Dulbecco’s phosphate-buffered saline (DPBS), DEPC water and penicillin–streptomycin (P/S) were purchased from Welgene (Gyeongsan, Korea). Fetal bovine serum (FBS) was provided by Atlas Biologicals (Fort Collins, CO, USA). Griess reagent, lipopolysaccharides from *Escherichia coli* O26:B6 (LPS), 3-(4,5-dimethylthiazol-2-yl)-2,5-diphenyl tetrazolium bromide (MTT), dimethyl sulfoxide (DMSO), sodium nitrite, skim milk powder and DNCB were purchased from Sigma Chemical Co. (St. Louis, MO, USA). RNAiso Plus was purchased from Takara Bio Inc. (Kusatsu, Japan). Chloroform, 2-propyl alcohol, acetone and olive oil were purchased from Daejung (Seongnam, Korea). iNOS, COX-2, IL-6, IL-1β and β-actin oligonucleotide coupled primers were synthesized by Integrated DNA Technologies (Coralville, IA, USA). An enzyme-linked immunosorbent assay (ELISA) kit for prostaglandin E_2_ (PGE_2_) was obtained from R&D Systems (Minneapolis, MN, USA), and an interleukin-6 (IL-6) ELISA kit was obtained from Abcam (Cambridge, UK). An interleukin-1β (IL-1β) ELISA kit was obtained from Invitrogen (Carlsbad, CA, USA). *TransScript*^®^ All-in-One First-Strand cDNA Synthesis SuperMix for qPCR (One-Step gDNA Removal) was purchased from TransGen Biotech Co. (Beijing, China). PowerSYBR^®^ Green PCR Master Mix from Applied Biosystems was purchased from Thermo Fisher Scientific (Rockford, IL, USA). P38, c-Jun N-terminal kinase (JNK), extracellular signal-regulated kinase (ERK), P65, phosphorylated P38 (p-P38), phosphorylated JNK (p-JNK), phosphorylated ERK (p-ERK) and phosphorylated P65 (p-P65) antibodies were purchased from Cell Signaling Technology (Danvers, MA, USA). All other reagents and materials were of the highest quality available.

### 2.3. Isolation of the Mentha Arvensis Essential Oil

The essential oil extracted from the whole plants of *Mentha arvensis* (MAEO) was obtained through steam distillation extraction (SDE). SDE has been used to separate hydrophobic substances, with a high boiling point that are insoluble in water. The dried plants (1 kg) were steam distilled at 100 °C for 90 min. Essential oil extraction was performed three times, and the yield (%) was estimated as the volume (mL) of oil obtained per 100 g of dried plants. The essential oil was dried over anhydrous sodium sulfate and stored at 4 °C. MAEO consists of a fat-soluble substance and was suspended in DMSO for further analysis.

### 2.4. Gas Chromatography-Mass Spectrometry (GC-MS) Analysis

GC-MS analysis of the MAEO was performed using a Varian CP3800 gas chromatograph combined with a Varian 1200 L mass detector (Varian, Inc., Palo Alto, CA, USA). The column mounted on the GC-MS was a VF-5MS capillary column treated with polydimethylsiloxane (30 m × 0.25 mm × 0.25 µm). The temperature of the oven was programmed at a rate of 5 °C/min from 50 °C to 250 °C. The ionization detector and injector temperatures were set at 200 °C and 250 °C. The carrier gas was injected with helium at a 1 mL/min constant flow rate. The 2 μL sample was injected with a split ratio of 10:1. An electronic ionization system with ionization energy of 70 eV was used in the mass spectrum. The scan was performed at 50–500 *m*/*z*. The component identification of the essential oil was based on the linear retention index of the GC peak for the homologous n-alkane series (C_8_–C_22_). The obtained mass spectra were compared with those reported in the National Institute of Standards and Technology (NIST, 3.0) library and the literature data [19]. Chemical standards containing menthol, menthone and piperitone were used as standards for this study because they are the main components of MAEO. For the quantitative determination of the major components from MAEO, these standard chemicals were purchased from Sigma Chemical Co. (St. Louis, MO, USA) with a purity of at least 90%. The standard solution diluted to an appropriate concentration was injected at 1.0 μL. The major components of MAEO were determined in triplicate. The calibration curves for the sample concentrations were plotted, and the major constituents in MAEO were selected based on their peak areas.

### 2.5. Cell Culture

RAW 264.7 cells (mouse-derived macrophages) were purchased from the Korean Cell Line Bank (KCLB, Korea). HaCaT cells (human epidermal keratinocyte) were provided by the food chemistry laboratory at Kangwon National University (Prof. OH Lee). The cells were cultured in Dulbecco’s modified Eagle’s medium (DMEM) with 100 units/mL penicillin–streptomycin (P/S) and 10% fetal bovine serum (FBS) at 37 °C and 5% CO_2_, followed by subculture every two to three days [20].

### 2.6. Cell Viability Analysis

Cell viability was measured to determine the cytotoxicity of MAEO using the MTT assay. Cultured cells were treated with MAEO for 24 h or stimulated with LPS (0.1 µg/mL) after 1 h. After incubation with MTT solution diluted 10:1 (5 mg/mL in PBS) at 37 °C for 4 h, purple formazan was formed in the cells. The solution in each well was completely removed, and then the purple formazan crystals were dissolved in DMSO and isopropyl alcohol at 1:1 (100 μL/well). The optical density was measured at 540 nm using a SpectraMax 190 microplate reader (Molecular Devices, San Jose, CA, USA).

### 2.7. Measurement of Nitric Oxide

RAW 264.7 and HaCaT cells were pretreated with MAEO at 12.5–100 μg/mL for 1 h, followed by stimulation with LPS (0.1 μg/mL) for 24 h. Nitrite accumulation in the culture medium as an indicator of NO production was measured using Griess reagent [21]. The culture supernatant (100 μL) was mixed with 100 μL of Griess reagent (equal volumes of 1% (*w*/*v*) sulfanilamide in 0.1% (*w*/*v*) naphthyl ethylenediamine-HCl and 5% (*v*/*v*) phosphoric acid) for 10 min [20]. The optical density was measured at 540 nm using a SpectraMax 190 microplate reader (Molecular Devices, San Jose, CA, USA). The amount of nitrite in the medium was determined with reference to a sodium nitrite (NaNO_2_) standard curve.

### 2.8. Enzyme-Linked Immunosorbent Assay (ELISA)

The levels of PGE_2_ (R&D Systems, Minneapolis, MN, USA), IL-6 (Abcam, Cambridge, UK) and IL-1β (Invitrogen, Carlsbad, CA, USA) in the culture medium were quantified using ELISA kits according to the manufacturer’s protocol.

### 2.9. RNA Isolation and Real-Time Polymerase Chain Reaction (RT-PCR)

RT-PCR was used to measure the mRNA expression of *iNOS*, *COX-2*, *IL-6* and *IL-1β*. Total RNA was extracted from RAW 264.7 macrophages and HaCaT cells using RNAiso PLUS. Total RNA (1 μg) was used to generate cDNA by reverse transcription using All-in-One First-Strand cDNA Synthesis SuperMix [22]. The synthesized cDNA was used as a template for qRT-PCR using QuantStudio 3 (Applied Biosystems, Foster City, CA, USA) system with FGPOWER SYBR Green PCR master mix and gene-specific primers (Table 1) [20,23]. PCR was carried out for 40 cycles under conditions of denaturation at 95 °C for 15 s, annealing at 57 °C for 20 s, and expansion at 72 °C for 40 s. A dissociation curve analysis of *iNOS*, *COX-2*, *IL-6*, *IL-1β* and *β-actin* mRNA showed a single peak. Expression levels of the target genes were quantified from duplicate measurements and normalized with the 2^−ΔΔCT^ method relative to *β-actin*.

### 2.10. Western Blot Analysis

For the Western blot analysis, the cells were washed twice with ice-cold phosphate-buffered saline (PBS) without calcium chloride or magnesium chloride. Then, the total proteins were isolated from the cells using lysis buffer (Jubiotech, Daejeon, Korea) after harvesting by scraping [20]. Total cellular protein (1 μg) was quantified using the Bradford assay [24]. The protein was resolved by 10% SDS-PAGE and transferred to PVDF membranes. The membranes were incubated with blocking buffer (5% skimmed milk powder in 1X TBS containing 0.1% Tween-20) for 2 h and then incubated with rabbit primary antibodies against phosphorylated forms of p-ERK (Cell Signaling Technology, 1:1000), p-P65 (Cell Signaling Technology, 1:1000), ERK (Cell Signaling Technology, 1:1000), P65 (Cell Signaling Technology, 1:1000), iNOS (Cell Signaling Technology, 1:1000), COX-2 (Cell Signaling Technology, 1:500), or β-actin (Cell Signaling Technology, 1:1000) overnight at 4 °C [22]. The membranes were washed and incubated for 2 h at room temperature (20 ± 5 °C) with the secondary antibody (Cell Signaling Technology, 1:1000). After washing, the membranes were developed using enhanced chemiluminescence. The immunoblots were imaged using a LAS-500 mini imager (General Electric, Boston, MA, USA) and analyzed using the ImageJ program, 1.51j8. The phosphorylation level was determined by calculating the ratio of the phosphorylated protein to the total protein on the same membrane using β-actin as the reference.

### 2.11. DNCB-Induced AD Mice

**Figure 1:**
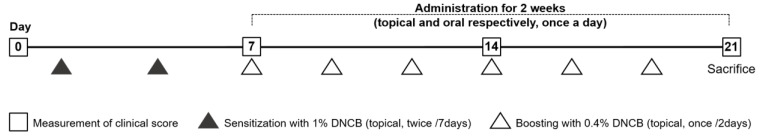
Experimental schedule for the DNCB-induced AD mouse experiment. Mice were sensitized with 1% DNCB twice a week. After sensitization, they were treated with MAEO (0.3%, 1%, topical), vehicle (olive oil, topical) or dexamethasone (1 mg/kg, p.o.) for 14 days.

Following the experimental schedule shown in Figure 1, the AD-like skin lesion mouse model was established. DNCB, a mouse allergic dermatitis-causing compound, was used to establish a murine model of atopic dermatitis. The dorsal skin of the mice was shaved with shaving cream and a clipper. The mice were divided into 5 groups (*n* = 5/group) as follows: the untreated group (Normal), the DNCB-sensitized group (Control), the treated group with MAEO 0.3% in olive oil (0.3%), the treated group with MAEO 1% in olive oil (1%) and the dexamethasone (1 mg/kg) treated group (positive control). To induce AD, DNCB was diluted to 1% in acetone and olive oil (3:1). The dorsal and ear skin of the mice were sensitized with 200 μL and 20 μL, respectively, of 1% DNCB twice a week. AD symptoms such as erythema, edema and papulation appeared in the DNCB treated dorsal and ear skin [7]. Seven days after shaving, 0.3% or 1% MAEO or olive oil was applied daily to the dorsal and ear skin for 2 weeks. Dexamethasone (1 mg/kg) was orally administered for 2 weeks. DNCB (0.4%) was applied once every 2 days to maintain the AD symptoms. The change in the AD clinical symptoms was measured by the dermatitis score every 7 days. The severity of the DNCB-induced AD lesions was evaluated according to the SCORing Atopic Dermatitis (SCORAD) index [25]. Scores from 0 (no lesion) to 3 (severe) were measured based on erythema, edema and papulation, excoriation and lichenification of the skin. The ear thickness was determined using a digital micrometer (Mitutoyo Co., Tokyo, Japan) on the last day of the experiment.

### 2.12. Histological Observation

On the final day, the dorsal skin tissue of each mouse was collected and fixed in 10% formalin solution at room temperature (20 ± 5 °C) and then embedded in paraffin [5,26]. Each section cut from the paraffin-embedded skin tissue was stained with hematoxylin and eosin (H&E) and toluidine blue (TB). Histological analysis and images were examined by light microscopy (Olympus, Tokyo, Japan). The epidermal thickness was analyzed by observing the portion stained with H&E at 100X magnification [27]. To evaluate the infiltration of mast cells, TB staining was performed, and the number of mast cells was counted in three randomly selected sections [28].

### 2.13. Statistical Analysis

All data analyses were performed using GraphPad Prism Version 8.0 (GraphPad, La Jolla, CA, USA). All measurements were expressed as the mean ± standard deviation (S.D.). All results were analyzed using the Student–Newman–Keuls test for multiple comparisons after one-way analysis of variance (ANOVA) was performed. Significance was defined as *p* < 0.05.

## 3. Results

### 3.1. GC-MS Analysis of MAEO

**Figure 2:**
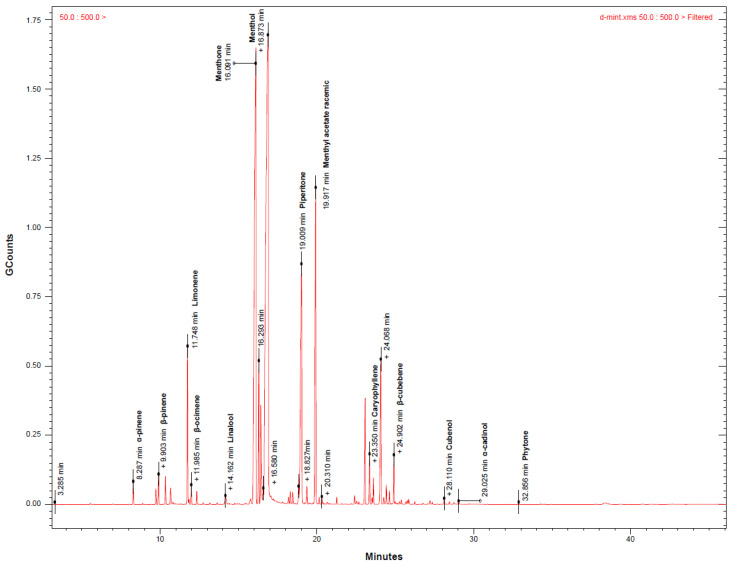
GC-MS Chromatogram of MAEO. MAEO contains chemical constituents corresponding to the 19 major peaks found in the GC-MS chromatogram. The main components of MAEO extracted using SDE are menthol and menthone.

MAEO, a transparent liquid with a spicy aroma, was obtained from whole plants. The yield of MAEO was 1.0580 ± 0.1106% (*v*/*w*) by the SDE. Figure 2 shows that the chemical composition of MAEO contained 19 major peaks found in the GC-MS chromatogram. In MAEO, a total of 49 different compounds were identified based on the retention time and mass spectral data (Table 2). The major components of MAEO were menthol (36.27%), menthone (25.71%) and piperitone (9.29%).

### 3.2. The Effects of MAEO on LPS-Induced Inflammatory Mediators and Proinflammatory Cytokines in RAW 264.7 Macrophages

**Figure 3:**
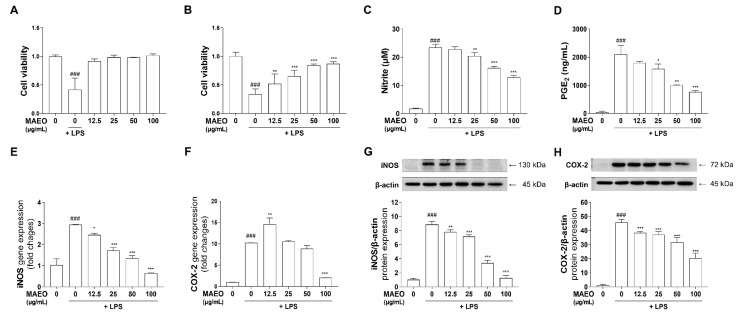
Effects of MAEO on cell viability and LPS-induced inflammatory response in RAW 264.7 macrophages. (**A**) Cytotoxicity and (**B**) cytoprotective effects induced with MAEO or LPS treatment were determined compared to 0 µg/mL MAEO. Production of (**C**) NO, (**D**) PGE_2_ were determined after LPS (0.1 µg/mL) stimulated for 24 h. The mRNA expression levels of (**E**) *iNOS*, (**F**) *COX-2*, and the fold changes are presented compared with 0 µg/mL MAEO. Relative protein expression of (**G**) iNOS, (**H**) COX-2 were also analyzed compared with the 0 µg/mL MAEO. *^###^ p* < 0.001 versus the 0 µg/mL MAEO; * p < 0.05, ** p < 0.01, *** p < 0.001 versus the LPS-stimulated group. The experiment was repeated at least three times and similar results were shown as mean ± S.D.

Macrophages play pivotal roles in the progression of atopic dermatitis through an immune response in the skin [29,30]. Therefore, we investigated whether MAEO exhibits anti-inflammatory effects on LPS-stimulated immune responses in RAW 264.7 cells. The cytotoxicity of MAEO was evaluated using MTT assays after incubating the cells with various concentrations of MAEO (0 to 100 μg/mL) for 24 h. No significant effect on cell viability was observed by MAEO treatment up to 100 μg/mL in RAW 264.7 cells (Figure 3A). Furthermore, it was confirmed that MAEO had a dose-dependent protective effect against LPS-induced apoptosis (Figure 3B). Therefore, we chose these concentrations for further studies. To investigate the effects of MAEO on LPS-induced inflammatory mediators (NO and PGE_2_), RAW 264.7 cells and the culture medium were pretreated with MAEO and stimulated with LPS (0.1 μg/mL) for 24 h. We observed that MAEO dose-dependently inhibited LPS-induced NO and PGE_2_ production, with IC_50_ values of 95.5 μg/mL and 67.4 μg/mL, respectively (Figure 3C,D). We also investigated whether MAEO affects the expression of the NO and PGE_2_ synthetic enzymes iNOS and COX-2. RT-PCR was performed to determine the mRNA levels of *iNOS* and *COX-2*. *iNOS* and *COX-2* mRNA expression was significantly increased in response to LPS, but MAEO especially inhibited these effects at 100 μg/mL (Figure 3E,F). Western blot analysis also revealed that the protein expression of iNOS and COX-2 were significantly increased by LPS treatment, which was counteracted by MAEO treatment in a dose-dependent manner (Figure 3G,H). These results indicate that MAEO treatment significantly suppressed LPS-induced NO and PGE_2_ production through inhibition of iNOS and COX-2 expression in RAW 264.7 cells. 

### 3.3. The Effects of MAEO on LPS-Induced Inflammatory Cytokines in RAW 264.7 Macrophages

**Figure 4:**
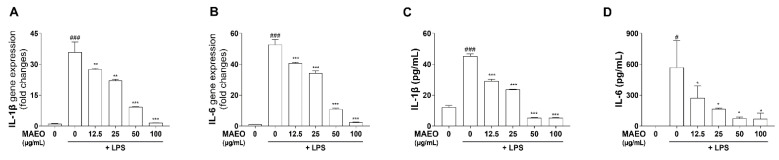
Effect of MAEO on LPS-induced IL-1β and IL-6 expression in RAW 264.7 macrophages. After stimulation with LPS (0.1 µg/mL) for 24 h, relative mRNA expression levels of (**A**) *IL-1β* and (**B**) *IL-6* were analyzed, and the fold changes are presented compared with the 0 µg/mL MAEO. Production of (**C**) IL-1β and (**D**) IL-6 were also determined. *^#^* *p* < 0.05, *^###^ p* < 0.001 versus the 0 µg/mL MAEO; * p < 0.05, ** p < 0.01, *** p < 0.001 versus the LPS-stimulated group. The experiment was repeated at least three times and similar results were shown as mean ± S.D.

It is well known that proinflammatory cytokines such as IL-6 and IL-1β play an important role in regulating inflammatory signals. Therefore, we further investigated the effects of MAEO on the expression of LPS-stimulated proinflammatory cytokines. By RT–PCR, we observed that LPS significantly increased the mRNA expression of *IL-6* and *IL-1β* compared to the control, but MAEO treatment dose-dependently suppressed *IL-1β* and *IL-6* mRNA expression (Figure 4A,B). Additionally, we found that MAEO significantly reduced the production of the inflammatory cytokines IL-1β and IL-6 (Figure 4C,D). These results suggest that MAEO has anti-inflammatory effects by inhibiting the expression of the proinflammatory cytokines IL-6 and IL-1β.

### 3.4. The Effects of MAEO on LPS-Induced ERK/NF-κB Activation in RAW 264.7 Macrophages

**Figure 5:**
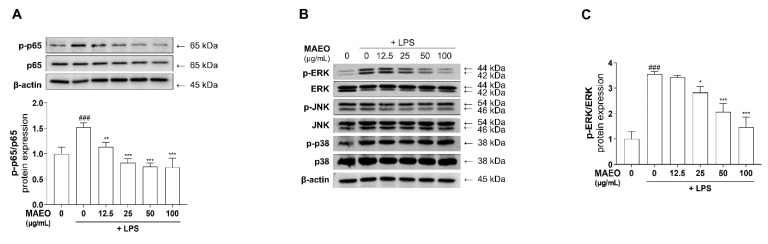
Effect of MAEO on LPS-induced ERK/NF-κB phosphorylation activity in RAW 264.7 macrophages. After stimulation with LPS (0.1 µg/mL) for 1 h, the immunoreactivity of (**A**) phosphorylated p65 (p-p65) and p65 and (**B**) phosphorylated ERK (p-ERK), JNK (p-JNK), p38 (p-p38) were analyzed. The relative protein expression levels of (**C**) p-ERK were quantified compared with 0 µg/mL MAEO. *^###^ p* < 0.001 versus the 0 µg/mL MAEO; * p < 0.05, ** p < 0.01, *** p < 0.001 versus the LPS-stimulated group. The experiment was repeated at least three times and similar results were shown as mean ± S.D.

In LPS-stimulated macrophages, the MAPK/NF-κB pathway is closely related to LPS-induced transcriptional regulation of inflammation [21,31]. To identify whether MAEO influences NF-κB activity, we measured the phosphorylation level of P65, a subunit of NF-κB, after MAEO treatment under LPS-induced stimulatory conditions. As shown in Figure 5A, the Western blots and quantitative results revealed that the increased phosphorylation of P65 protein induced by LPS was dose-dependently reduced by MAEO treatment, whereas the total amount of P65 was unchanged by any treatment. Since NF-κB activity is regulated by MAPKs, the activities of ERK, JNK and P38 were checked using Western blots. After pretreatment with MAEO, phosphorylated ERK, but not JNK and P38, was effectively decreased by MAEO treatment (Figure 5B,C). These results indicate that the anti-inflammatory activity of MAEO was caused by the suppression of the ERK-NF-κB signaling pathway.

### 3.5. The Effects of MAEO on LPS-Induced Inflammatory Responses in HaCaT Human Keratinocytes

**Figure 6:**
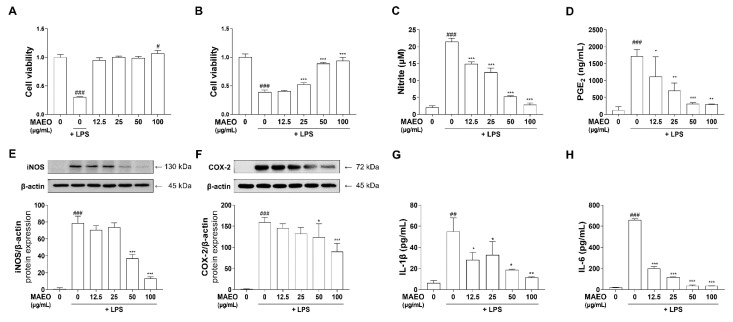
Effect of MAEO on cell viability and anti-inflammatory properties in HaCaT keratinocytes. (**A**) Cytotoxicity and (**B**) cytoprotective effects induced with MAEO or LPS treatment were determined compared to 0 µg/mL MAEO. Production of (**C**) NO, (**D**) PGE_2_ were determined after LPS (0.1 µg/mL) stimulated for 24 h. Relative protein expression of (**E**) iNOS, (**F**) COX-2 were analyzed compared with the 0 µg/mL MAEO. Production of (**G**) IL-1β and (**H**) IL-6 were also determined. *^#^ p* < 0.05, *^##^ p* < 0.01, *^###^ p* < 0.001 versus the 0 µg/mL MAEO; * p < 0.05, ** p < 0.01, *** p < 0.001 versus the LPS-stimulated group. The experiment was repeated at least three times and similar results were shown as mean ± S.D.

Allergies, AD and other skin diseases are caused by excessive inflammation, a type of innate immune response, in the skin. Since keratinocytes are most closely related to the immune response, we tested the anti-inflammatory effect of MAEO in HaCaT cells, a human-derived keratinocyte, under LPS-induced inflammatory conditions. To investigate the effect of MAEO on the LPS-induced inflammatory response in human keratinocytes, HaCaT cells were pretreated with MAEO for 1 h and then stimulated with LPS (0.1 μg/mL) for 24 h. Similar to the results in macrophages, no significant cytotoxicity was found in HaCaT cells after MAEO treatment (0 to 100 μg/mL) for 24 h or stimulated with LPS (0.1 µg/mL) after 1 h (Figure 6A,B). Furthermore, LPS upregulated the production of inflammatory mediators such as NO and PGE_2_, which were reduced dose-dependently by MAEO treatment (Figure 6C,D). Moreover, the protein expression of iNOS and COX-2, the synthetic enzymes of NO and PGE_2_, was also decreased by MAEO treatment (Figure 6E,F). In addition, MAEO treatment also reduced IL-6 and IL-1β production after LPS exposure (Figure 6G,H). Thus, MAEO has anti-inflammatory effects on LPS-stimulated immune responses in human keratinocytes.

### 3.6. The Effects of MAEO on the DNCB-Induced AD Animal Model

**Figure 7:**
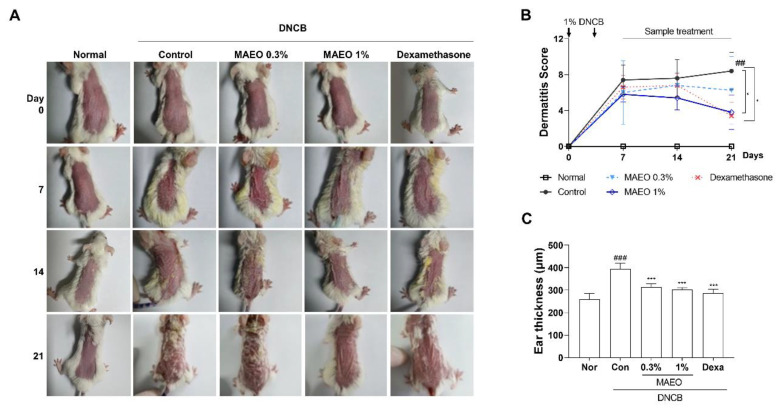
Effect of MAEO on DNCB-induced AD-like skin lesions in BALB/c mice. (**A**) Images of the dorsal skin during the experiment were acquired at the same magnification and representative images are shown. (**B**) Dermatitis score was measured every 7 days and (**C**) ear thickness was measured at 21 day. The results are presented as the mean ± S.D. *^##^ p* < 0.01, *^###^ p* < 0.001 versus the normal group, * p < 0.05, *** p < 0.001 versus the control group. Nor; normal, Con; control, Dexa; dexamethasone.

Based on the in vitro studies, we hypothesized that MAEO would have anti-inflammatory activity against skin disease, particularly AD. To investigate the effects of MAEO on AD, we employed a DNCB-induced murine model of AD. The clinical symptoms of the DNCB-induced AD model were determined by the ear thickness and the SCORAD index on the dorsal skin at intervals of 7 days. As shown in Figure 7A, topical treatment with 1% MAEO for 14 days dramatically improved the AD symptoms compared to the vehicle-treated controls. The SCORAD index of the control group was significantly increased compared to that of the normal group. However, topical treatment with 1% MAEO decreased the SCORAD index, and surprisingly, this effect was similar to that of the positive control dexamethasone (Dexa) (Figure 7B). In addition, MAEO significantly reduced the DNCB-induced increase in ear thickness compared to the controls (Figure 7C). These results indicate that topical treatment with MAEO could effectively attenuate the clinical symptoms of AD.

**Figure 8:**
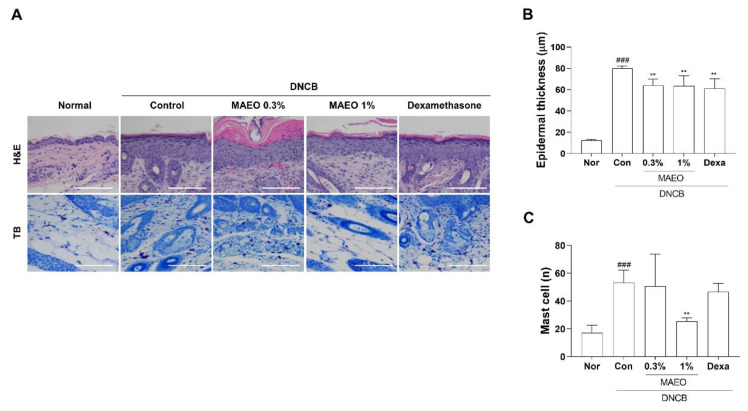
Effect of MAEO on DNCB-induced AD-like skin lesions in BALB/c mice. (**A**) Images of hematoxylin and eosin (H&E) and toluidine blue (TB) staining of the dorsal skin were acquired at 200× magnification, and representative images are shown, scale bar: 100 μm. (**B**) Epidermal thickness and (**C**) number of infiltrated mast cells were calculated, and the results are presented as the mean ± S.D. *^###^* *p* < 0.001 versus the normal group, ** p < 0.01 versus the control group. Nor; normal, Con; control, Dexa; dexamethasone.

To investigate the histological effects of MAEO on DNCB-induced epidermal thickness and mast cell infiltration in dorsal skin tissue, H&E and TB staining were performed (Figure 8). In a DNCB-induced murine model of AD, the average epidermal thickness and the number of infiltrated mast cells were remarkably increased. However, the DNCB-induced epidermal thickness in dorsal skin was significantly reduced by topical treatment with 0.3% and 1% MAEO compared to the vehicle-treated controls (Figure 8B). In addition, the invasion of mast cells was also remarkably reduced by treatment with 1% MAEO to a level similar to that of the normal group (Figure 8C). These findings suggest that the anti-inflammatory properties of MAEO may lead to anti-atopic dermatitis in a DNCB-induced animal model of AD.

## 4. Discussion

In the present study, we demonstrated the anti-inflammatory effects of MAEO and its underlying molecular mechanisms in mouse-derived macrophage RAW 264.7 cells and human-derived keratinocyte HaCaT cells. Furthermore, the anti-atopic dermatitis effects of MAEO were confirmed in a DNCB-induced AD-like animal model. Dysregulated production of inflammatory mediators, such as NO, PGE_2_ and proinflammatory cytokines, is involved in the inflammatory response in AD [32]. NO and PGE_2_ are synthesized by iNOS and COX-2 enzyme, respectively, and they induce inflammatory symptoms such as fever, edema, and pain [12,13,33]. Additionally, proinflammatory cytokines such as IL-6 and IL-1β transmit inflammatory signals, which significantly impact immune and inflammatory response regulation [34]. Thus, the reduction of inflammatory mediators such as NO, PGE_2_, and cytokines is important for the prevention of inflammatory disease [35]. In this study, MAEO suppressed the LPS-induced increase in NO and PGE_2_, and their synthetic enzymes, iNOS and COX-2 in RAW 264.7 macrophages. Notably, MAEO showed a different decrease tendency between mRNA and protein of COX-2. We speculate that MAEO may have its downstream effects after mRNA expression that influences post-transcriptional processes, such as interference with protein folding of COX-2. Furthermore, a previous study supported that the expression relationship between mRNA and protein may differ from the ideal under various stressful situations [36]. In addition, MAEO reduced the mRNA expression and production of proinflammatory cytokines IL-6 and IL-1β. Similar to the results from RAW 264.7 cells, the anti-inflammatory effects of MAEO were also observed in the LPS-stimulated inflammatory response in HaCaT cells. Keratinocytes play a key role in innate immunity and pathogen detection. Host defense in the skin is carried out through the expression of numerous pattern recognition receptors and the production of various inflammatory mediators that respond to the stimulation of pathogens [37]. This led us to speculate that MAEO could modulate AD by inhibiting inflammation in HaCaT, a human-derived keratinocyte cell line.

AD, a chronic inflammatory disease, is usually caused by an imbalance in T helper (Th) 1/Th2 cells, and immune cells such as macrophages and mast cells are recruited to the lesions [38,39]. These factors cause abnormal apoptosis regulation of keratinocytes, leading to the collapse of the epithelial structure [40]. This causes increased epidermis thickness, edema, erythema, and lichenized plaques in AD [41]. BALB/c mice induced by AD using DNCB were suggested to exhibit symptoms similar to human AD, such as hemorrhage, epidermal hyperplasia, mast cell infiltration and increased immunoglobulin E (IgE) levels in the serum [42]. Based on the anti-inflammatory action of MAEO, we evaluated the anti-AD effects of MAEO in vivo. In this study, AD skin lesions induced using repeated application of DNCB to the ears and dorsal skin of mice were treated with MAEO. The relief of AD symptoms by 1% MAEO was visually observable, and the consistently increased ear thickness was significantly decreased compared to the control group. Moreover, the effects of topical MAEO administration were shown by significant reductions in the epidermal thickness of the dorsal skin and infiltration of mast cells.

MAEO exhibits a wide range of biological and pharmacological activities [43]. MAEO with 49 chemicals mainly contains menthol (30–43%), menthone (18–32%), and menthyl acetate (10–30%) [44]. Menthol, the major component of MAEO, was reported to have various effects, such as antibacterial, analgesic and antitumor effects [45,46]. Menthone has been widely used as a cooling agent and a counterirritant for pain relief [47]. Actually, similar ingredients were also reported in the essential oil extracted from *Mentha piperita*, a plant of the same genus as MAEO, that exerts the strong anti-inflammatory effects in the LPS-induced RAW 264.7 cells and the croton oil-induced mouse ear edema model [48]. Additionally, antioxidant, anti-inflammatory and cytoprotective properties were reported in ethanol extracts of three species of mint (*Mentha spicata* L., *Mentha pulegium* L. and *Mentha rotundifolia* (L). Huds [49]. Therefore, MAEO is expected to have excellent anti-inflammatory and pain relief effects as it is rich in menthol and menthone. Taken together, these results indicate that topical treatment with MAEO could be a therapeutic option for AD.

**Figure 9:**
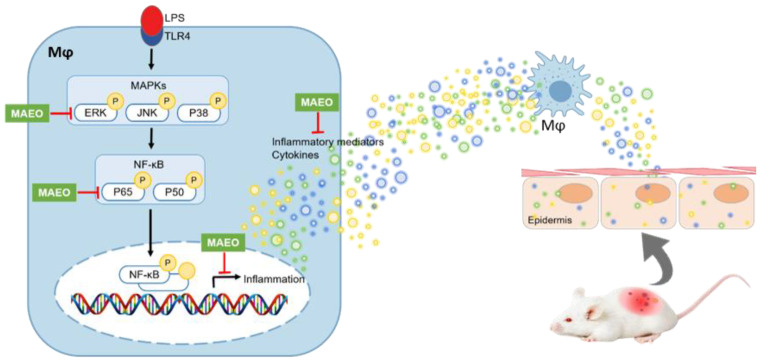
Proposed mechanism of action of MAEO under LPS-induced inflammatory and DNCB-induced AD-like conditions.

In this study, we observed that MAEO reduced the phosphorylation of ERK and P65 in LPS-induced RAW 264.7 macrophages (Figure 9). As a representative NF-κB, P65 is activated to induce various cellular responses, tissue repair, and inflammation-related genes. Previous studies have shown that MAPK and NF-κB pathway activation is the first signal to transcriptionally upregulate chemokines and cytokines when LPS stimulates macrophages [50]. ERK, a member of the MAPK family, is activated by not only Toll-like receptors (TLRs) that recognize LPS but also Janus kinase (JAK)-signal transducer and activator of transcription (STAT) signaling of cytokine receptors [51]. LPS-induced cytokines are recognized as cytokine receptors by the surrounding cells. Importantly, IL-6 has the strongest effect on the phosphorylation of STAT3, which triggers a cytokine storm leading to various chronic inflammatory diseases [52,53,54]. Although the expression and function of NF-κB in chronic inflammatory skin diseases such as AD is still unclear, there are many reports that it is related to the inflammatory response [31,55,56]. These data suggest that activation of inflammation may play a crucial role in chronic inflammatory skin diseases. All of these findings support the anti-inflammatory effects of MAEO found in previous studies. Therefore, MAEO might suppress the ERK-NF-κB pathway and effectively reduce excessive inflammation and the subsequent AD-like lesions.

In conclusion, we confirmed the effects of MAEO on LPS-induced inflammatory stimulation in RAW 264.7 macrophages and HaCaT keratinocytes. As reported, menthol and menthone are the main ingredients of MAEO and are thought to have anti-inflammatory properties [44]. Furthermore, MAEO might be a promising treatment for systemic inflammatory disease, especially in AD due to its activity in inhibiting the ERK/NF-κB pathway.

## Figures and Tables

**Table 1:** Primer sequences for RT-PCR.

Target Gene	Primer Sequence		Accession Number
*iNOS*	F	5′-CAT GCT ACT GGA GGT GGG TG-3′	NM_010927
	R	5′-CAT TGA TCT CCG TGA CAG CC-3′	
*COX-2*	F	5′-TGC TGT ACA AGC AGT GGC AA-3′	NM_011198
	R	5′-GCA GCC ATT TCC TTC TCT CC-3′	
*IL-6*	F	5′-GAG GAT ACC ACT CCC AAC AGA CC-3′	NM_031168
	R	5′-AAG TGC ATC ATC GTT GTT CAT ACA-3′	
*IL-1β*	F	5′-ACC TGC TGG TGT GTG ACG TT-3′	NM_008361
	R	5′-TCG TTG CTT GGT TCT CCT TG-3′	
*β-Actin*	F	5′-ATC ACT ATT GGC AAC GAG CG-3′	NM_007393
	R	5′-TCA GCA ATG CCT GGG TAC AT-3′	

**Table 2:** Composition of MAEO.

Compound Name		Retention Time	Area (%)
1	Toluene	3.285	0.04
2	α-Pinene	8.287	0.43
3	β-Phellandrene	9.733	0.28
4	β-Pinene	9.903	1.07
5	3-Octanol	10.666	0.33
6	Limonene	11.748	3.06
7	Eucalyptol (Cineole)	11.846	0.06
8	β-Ocimene	11.985	0.32
9	α-Pinene	12.336	0.23
10	Linalool oxide	13.163	0.04
11	Terpinolene	13.634	0.04
12	Linalool	14.162	0.26
13	Menthone	16.091	25.71
14	Menthol	16.422	2.4
15	isopulegone	16.580	0.29
16	Menthol	16.873	36.27
17	2,3-Dimethyl-2-cyclopenten-1-one	18.189	0.12
18	Hexen-3-yl Valerate	18.292	0.21
19	Pulegone	18.452	0.21
20	Piperitone oxide	18.827	0.44
21	Piperitone	19.009	9.29
22	Menthol	19.356	0.38
23	Menthyl acetate racemic	19.917	8.3
24	Diosphenol	20.141	0.1
25	D-Verbenone	21.258	0.12
26	β-Bourbonene	22.398	0.15
27	β-Elemene	22.529	0.05
28	Jasmone	22.670	0.04
29	p-Menthane-1,2,3-triol	23.064	1.94
30	Caryophyllene	23.350	0.88
31	p-Menthane-1,2,3-triol	23.516	0.12
32	1-Acetoxy-p-menth-3-one	23.601	0.45
33	3,5,5-Trimethylbicyclo[4.1.0]heptan-2-one	24.068	3.78
34	α-Caryophyllene	24.261	0.12
35	Bicyclosesquiphellandrene	24.420	0.31
36	β-Cubebene	24.902	0.84
37	Rose butanoate	24.990	0.03
38	Elixene	25.258	0.05
39	Butylated Hydroxytoluene (BHT)	25.379	0.08
40	γ-Cadinene	25.675	0.05
41	β-Cadinene	25.779	0.07
42	Calamenene	25.854	0.09
43	α-Murolene	26.236	0.05
44	1,7-Dimethyl-4-propan-2-ylcyclodeca-2,7-dien-1-ol	27.204	0.07
45	Caryophyllene oxide	27.355	0.03
46	Cubenol	28.110	0.11
47	Benzeneacetic acid	28.436	0.04
48	α-Cadinol	29.025	0.08
49	Phytone	32.856	0.04
Total		99.47	
Yield (*v*/*w*, %)		1.06	

## Data Availability

Data is contained within the article.

## Abbreviations

AD: atopic dermatitis; COX-2, cyclooxygenase-2; Dexa, dexamethasone; DNCB, dinitrochlorobenzene; ERK, extracellular signal-regulated kinase; IgE, Immunoglobulin E; IL, interleukin; iNOS, inducible nitric oxide synthase; JAK, Janus kinase; JNK, c-Jun N-terminal kinase; LPS, lipopolysaccharide; MAEO, *Mentha arvensis* essential oil; MAPK, mitogen-activated protein kinase; NF-κB, nuclear factor-kappa B; NO, nitric oxide; PGE_2_, prostaglandin E_2_; STAT, signal transducer and activator of transcription; Th, T helper; TLRs, Toll-like receptors; TNF-α, tumor necrosis factor-α
